# Highly efficient antibiofilm and antifungal activity of green propolis against *Candida* species in dentistry materials

**DOI:** 10.1371/journal.pone.0228828

**Published:** 2020-12-23

**Authors:** Carolina Rabelo Falcão Bezerra, Katia Regina Assunção Borges, Rita de Nazaré Silva Alves, Amanda Mara Teles, Igor Vinicius Pimentel Rodrigues, Marcos Antonio Custódio Neto da Silva, Maria do Desterro Soares Brandão Nascimento, Geusa Felipa de Barros Bezerra

**Affiliations:** 1 Post-Graduate Program in Adult Health, Federal University of Maranhão – UFMA, São Luís, Brazil; 2 Post-Graduate Program in Adult Health, Basic and Applied Immunology Nucleum (NIBA/DEPAT), Federal University of Maranhão – UFMA, São Luís, Brazil; 3 Post-Graduate Program in Biotechnology, Federal University of Maranhão – UFMA, São Luís, Brazil; 4 Post-Graduate Program in Internal Medicine, State University of Campinas (UNICAMP), Campinas, Brazil; Lebanese American University, LEBANON

## Abstract

This study evaluated the effect of green propolis extract on the adhesion and biofilm formation of *Candida* species in dentistry materials. Phytochemical analysis of green propolis extract was performed by high-performance liquid chromatography. Adhesion was quantified by counting the number of yeast cells adherent to dental material fragments in a Neubauer chamber. Biofilm formation was determined by counting colony-forming units recovered from dental material fragments. The intensity of biofilm adhesion was classified as negative, weak, moderate, strong, or very strong. Fifteen compounds, mainly flavonoids, were identified in green propolis extract. All strains adhered to and formed biofilms on the surfaces of the orthodontic materials studied. On steel and resin, yeast cell adhesion intensities were weak at all incubation times, except for those of *Candida parapsilosis* and *C*. *tropicalis*, which were moderate at 12 h. At 24 and 48 h, *C*. *albicans* formed biofilms on steel with moderate adhesion affinities; at 24 and 48 h, *C*. *parapsilosis* formed biofilms with very strong affinities. *C*. *tropicalis* formed biofilms with strong and very strong affinities at 24 and 48 h, respectively. On resin, all species displayed strong affinity for biofilm formation at 24 and 48 h, except for *C*. *tropicalis*, which displayed very strong affinity at only 48 h. Green propolis extract displayed antifungal activity and inhibited both adhesion and biofilm formation at 2.5 μg/mL. This study reinforces the idea that green propolis has antifungal activity and interferes with the virulence of *Candida* species.

## Background

Recent years have seen increased use of orthodontic materials for aesthetic, surgical, and biofunctional purposes. Polymers, ceramics, composites, resins, steel, and steel alloys are used to manufacture dental prostheses, screws, and orthodontic appliances. When implanted into the oral cavity, they are exposed to colonization and biofilm formation by microorganisms that live in the oral cavity. Saliva and oral pH facilitate the targeting of these devices for biofilm formation, especially by *Candida* spp. [1].

Candida are normal commensal organisms in the mouth that most frequently populate the posterior part of the dorsum of the tongue. They can also be found on other body surfaces, in the vagina, and in the digestive tract [2, 3].

A combination of factors contributes to *Candida* spp. colonization and biofilm formation, including salivary flow, low pH, poor oral hygiene, and type of orthodontic material [2]. During colonization and biofilm formation, oral microbiotasecrete enzymes and exopolysaccharides to colonize a surface. Their biofilms consist of a film of organic components that form an extracellular polymeric matrix that incorporates a multicellular microbial community (bacteria and/or fungi) [4–6].

Formation of biofilms on orthodontic materials raises concerns because once established, they increase the systemic risk of infection and antibiotic and antifungal resistance, becoming a beachhead of infection and an obstacle to effective therapy. Natural products may inhibit biofilm formation; however, antibiofilm effects depend on the inhibition of extracellular matrix formation, adhesin inhibition, cell attachment inhibition, and inhibition of virulence factors [6].

Propolis is a natural product resin with medicinal properties. Propolis is produced by mixing a collection of plant structures with wax and bee salivary enzymes. It functions in the hive as a varnish, protecting and disinfecting the internal and external hive surfaces and maintaining constant humidity and temperature [5–8].

Brazil has at least 13 distinct types of propolis that cumulatively contain many bioactive compounds, including apigenin, artepillin C, vestitol, and neovestitol [9]. Some varieties of propolis, namely red, green, yellow, and brown propolis, are distinguished by their flowering period. Green propolis is usually obtained as a sticky exudate from the leaves, flower buttons, buds, stems, and fruits of *Baccharis dracunculifolia* [10]. This substance is rich in compounds with antimicrobial properties, such as prenylated phenylpropanoids, triterpenoids, and benzoic and chlorogenic acids [11–13].

The use of propolis in dentistry is increasing. It has been used clinically for gingivitis, dental caries, oral candidiasis, oral herpes, and other diseases [14]. In addition to dental applications, Brazilian green propolis has several other biological properties, including anti-inflammatory [15], antihypertensive [16], antihyperlipidemic [17], antioxidant [18], and antitumor [19] effects. Recently, it has been used as a neuroprotectant against neurodegenerative diseases [20].

It has been reported in scientific literature that green propolis has antifungal and antibacterial activities against *Lasiodiplodia theobromae* [21], *Candida* spp. [22], *Streptococcus mutans* [23], *Streptococcus acidominimus*, *Streptococcus oralis*, *Staphylococcus epidermidis*, *Veillonella parvula*, *Bifidobacterium breve*, *Bifidobacterium longum*, and *Lactobacillus acidophilus* [24].

Several studies have shown the fungistatic and fungicidal effects of propolis in different species of yeast of the genus *Candida*, both *in vitro* and *in vivo*. *Candida albicans* is relatively pathogenic and is the predominant *Candida* species found in candidiasis lesions of the oral mucosa. However, the proportion of other species, such as *C*. *tropicalis*, *C*. *krusei*, *C*. *parapsilosis*, and *C*. *guilliermondii*, increases in these lesions over the course of the disease. This pathology is often found in the elderly (especially in patients with prostheses), young children, patients with diabetes, and those who have undergone prolonged immunosuppression therapy (pharmacologically or owing to human immunodeficiency virus/acquired immunodeficiency virus) or prolonged use of antibiotics [25–27].

In addition to propolis, several medicinal plants from the order *Lamiales*, *Apiales*, *Asterales*, *Myrtales*, *Sapindales*, *Acorales*, *Poales*, and *Laurales* have been reported to inhibit *Candida* biofilms. Chemical compounds such as flavonoids, terpenoids, saponins, and alkaloids have been shown to be responsible for this antimicrobial property [28].

The aim of this study was to evaluate the effects of green propolis extract on the virulence factors (adhesion and biofilm formation) of *C*. *albicans*, *C*. *tropicalis*, and *C*. *parapsilosis* on dental materials (acrylic resin and steel).

## Materials and methods

### Preparation of green propolis ethanolic extract (EEPV)

The green propolis used in the described *in vitro* assays was acquired from Rosita Apiary (Betim-MG). Raw propolis was stored in a dry, airless plastic bag and refrigerated until used. The hydroalcoholic extract of green propolis was obtained according to the methods of Soares de Moura et al. [29]. Approximately 200 g of green propolis was diluted in 500 mL of PA ethyl alcohol and stored at room temperature in an amber flask with stirring (2 h/day) for 8 days. It was then filtered and evaporated at 35°C until the solvent was completely removed. The resulting concentrate was lyophilized and refrigerated until used.

### Phytochemical screening

The extract was subjected to phytochemical screening using the methods described by Matos [30] to detect phenols and tannins (reaction with ferric chloride); anthocyanins, anthocyanidins, flavonoids, leucoanthocyanidins, catechins, and flavanones (pH variation using hydrochloric acid and sodium hydroxide); and flavanols, flavanones, flavanonols, and xanthones (reaction with metallic magnesium and concentrated hydrochloric acid). The results obtained in each test were qualitatively evaluated by staining and precipitation reactions.

### Determination of total phenolic content

The total phenolic content of the extract was determined by the Folin–Ciocalteu method based on the procedures described by Waterhouse [31], with some modifications. In this case, we used tannic acid instead of gallic acid, and the concentrations tested were different.

For standard curve determination for tannic acid, a 2,000 μg.mL^-1^ solution was prepared and used to produce five dilutions (10, 25, 50, 75, 100, and 125 μg mL^-1^ tannic acid). Thereafter, 500 μL of each solution was diluted in 2.5 mL of 10% (v/v) Folin–Ciocalteu solution, and mixed with 2 mL of 4% (v/v) sodium carbonate solution in test tubes. These mixtures were protected from light. After 30 min, the absorbance was read on a spectrophotometer at 760 nm using a quartz cuvette. Absorbance readings were plotted as a function of tannic acid concentration using the regression equation and its coefficients [31].

### Evaluation of antioxidant activity by 2,2-diphenyl-1-picrylhydrazly

The antioxidant activity of the extracts was evaluated using 1,1-diphenyl-2-picrilidrazil (DPPH), according to the methods described by Yen and Wu [32]. For a range of extract concentrations (10, 25, 50, 75, 100, 125, 150, 175, 200, and 225 μg/mL), reaction mixtures with DPPH were prepared. One milliliter of each dilution was transferred to a test tube containing 3.0 mL of DPPH ethanolic solution (0.004%). After 30 min of incubation in the dark at room temperature, DPPH free radical reduction was measured by reading the absorbance at 517 nm using a spectrophotometer. A blank sample was prepared using ethanol instead of extract. Eq 1 was used to calculate sequestration of free radicals expressed as a percentage of radical oxidation inhibition.

Antioxidantactivity(%)=[1–(sampleabsorbance/controlabsorbance)×100. (1)

IC_50_ values (concentration of extract required to sequester 50% of DPPH radicals) were calculated using the above equation based on the concentration of each extract and its respective percentage of DPPH radical sequestration.

These analyses were performed at the Chemical Research Laboratory of the Federal University of Maranhão.

### Analysis of phytochemical composition

The phytochemical composition of the extract was analyzed by high-performance liquid chromatography (HPLC) coupled to mass spectrometry (HPLC-DAD-MS). Chromatographic analyses were performed at the Instrumentation Analytical Center of the Institute of Chemistry of the University of São Paulo. After solubilization, samples of green propolis hydroalcoholic extract were analyzed by HPLC. A Shimadzu^®^ chromatograph (Shimadzu Corp. Kyoto, Japan) comprising a solvent injection module with a Shimadzu LC-20AD pump and a Shimadzu UV-Vis detector (SPDA-20A) was used for analysis. The column used was a Supelco Ascentis C-18 (250 × 4.6 mm; 5 μm). HPLC was performed with an elution gradient using a mobile phase containing 5% acetic acid in varying proportions of water and methanol (organic phase). The total run time was 115 min. The injection volume was 20 μL, and chromatographic acquisition was performed at 270 nm (DAD). Data were collected and processed using the LC Solution software (Shimadzu). Identification of compounds by mass spectrometry was performed in the negative mode.

### Dental materials and microorganisms

Fragments of self-curing acrylic (Resin, Dêncor^®^) and Orthodontic Band (Metal, Morelli^®^) dental materials were purchased from dental shops. Three species of *Candida* were used in this study: *C*. *albicans* ATCC 443-805-2, *C*. *parapsilosis* ATCC 726-42-6, and *Candida tropicalis* ATCC 1036-09-2 were obtained from the stock collection of the Collection of Fungi of Immunology and Mycology Laboratory—NIBA/UFMA.

### Evaluation of EEPV antifungal activity

Initially, *Candida* species were cultivated on Sabouraud agar incubated at 37°C in a BOD greenhouse. After 24 h, each species was diluted in saline to a turbidity of 0.05 on the McFarland scale. Antifungal activity was assessed by disk diffusion method on Mueller-Hinton agar with 2% dextrose and 0.5 μg/mL methylene blue, as recommended by the CLSI M44-A2 protocol [33], with some modifications for natural products [34, 35] Amphotericin B was diluted in 1× PBS plus 1% DMSO to a concentration of 16 μg/mL as a positive control. To evaluate antifungal activity, 50 mg of EEPV was diluted in 500 μl of DMSO. A working solution was prepared by diluting 1 ml of this stock in 9 ml of 1× PBS. From this working solution, extract concentrations of 0.25, 2.5, 25, and 250 μg/mL were prepared.

The cut-off levels of susceptibility to amphotericin were utilized according to CLSI supplement M27-S3 [33], and that to propolis was used according to Silici and Koc [34] to identify strains as susceptible (S), dose-dependent susceptible (DDS), and resistant (R) (Table 1).

**Table 1:** Interpretation criteria for fungal susceptibility to amphotericin B by disk diffusion assay.

Substance	Susceptible	Dose-dependent susceptibility by disk diffusion assay	Resistant μg/mL
Amphotericin B	>10 mm	-	≤10 mm

The minimum inhibitory concentration (MIC) for propolis was defined as the lowest concentration in which optical clarity was observed [34, 35].

### Adherence and biofilm formation on abiotic and acrylic resin surfaces

Five centimeter-diameter fragments of dental material (metal or acrylic resin) were generated as described by Silva et al. [36] and Borges et al. [37] with modifications. These fragments were cultivated in 100 μl of saline containing a 1×10^4^ cell/mL suspension of *C*. *albicans*, *C*. *parapsilosis*, or *C*. *tropicalis* and kept in a BOD greenhouse for 3, 6, or 12 h for adherence assays or for 24 and 48 h for biofilm formation assays. All assays were performed in triplicate. After incubation with *Candida* species, the fragments were washed with sterile distilled water thrice, fixed with PA alcohol, and stained with crystal violet. Subsequently, the fragments were added to tubes containing 3 mL of 0.85% saline and vortexed for 10 min to obtain a suspension of fungal cells adherent to the materials. Ten microliters of adherence test suspension was added to a Neubauer chamber for counting of adherent cells by light microscopy. The strength of adhesion to a dental material was based on the counts and classified into the following groups: negative: <50 yeast/ml; weak: between 50 and 499 yeast/ml; moderate: 500 to 999 yeast/ml; and strong: 1000 or more yeast/ml. For the biofilm test, 100 μl of suspension was added to a plate containing Mueller-Hinton agar to quantify the number of colony-forming units (CFUs). The strength of biofilm formation on a dental material was classified into the following groups: negative: without CFU; weak: between one and 199 CFUs; moderate: 200 to 499 CFUs; strong: 500 to 1000 CFUs; and very strong: over 1000 CFUs.

### Antiadherence and antibiofilm activities of EEPV

EEPV dilutions (0.25, 2.5, 25, and 250 μg/mL) were prepared as described above. To evaluate the effect of each dilution, fragments were cultivated in a tube containing 3 mL of each concentration of EEP and incubated in a BOD greenhouse at 37°C for 3, 6, and 12 h for adhesion, and for 24 and 48 h for biofilm formation. After each period, tubes were removed from the greenhouse, and fragments were washed thrice with sterile distilled water. After washes and greenhouse drying, the fragments were fixed with PA ethyl alcohol and stained with crystal violet. The fragments were then added to a tube containing saline and vortexed for 10 min.

### Statistical analysis

Data were analyzed using the GraphPad Prism R version 7 software. Two-way analysis of variance with Tukey’s *post-hoc* test was performed, where p <0.05 and confidence interval of 95% were considered significant.

## Results

### Phytochemical screening

In the present study, the extract showed strong reactivity, which indicated the presence of flavones, flavonoids, and xanthones. The average intensity of reactions indicating the presence of alkaloids, condensed tannins, and hydrolysable tannins is shown in Table 2.

**Table 2:** Classes of secondary metabolites identified in green propolis extract.

Class of metabolite	Presence in the hydroalcoholic extract of green propolis
Phenols	+
Alkaloids	++
Condensed tannins	++
Hydrolysable tannins	++
Anthocyanins and anthocyanidins	-
Flavones, flavanols, and xanthones	+++
Chalcones and aurones	-
Leucoanthocyanidins	-
Catechins	-
Flavanones	++
Free steroids	
Free Pentacyclic Triterpenoids	++
Saponins	—

Key: Strong (+++), medium (++), weak (+), and absent (-) reactions.

### Chemical composition of green propolis hydroalcoholic extract by HPLC-DAD-MS

**Fig 1:**
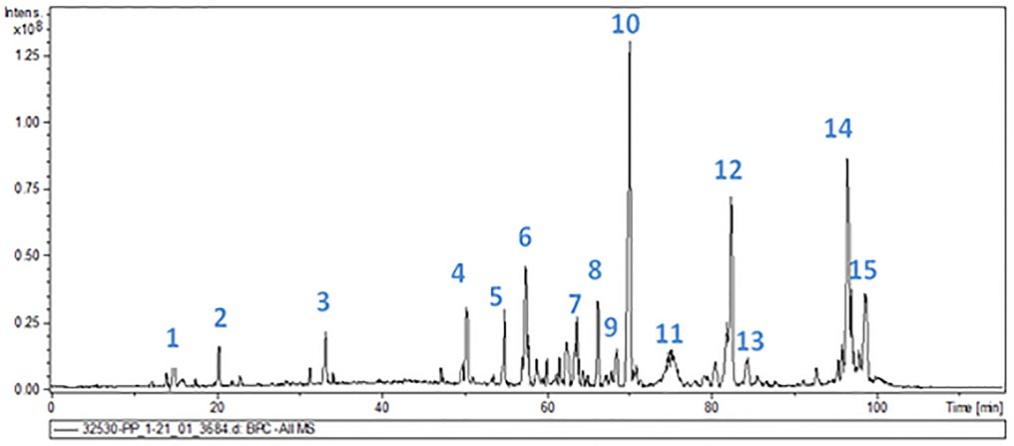
Chromatogram of the hydroalcoholic green propolis extract monitored by absorbance at 270 nm.

The compound profile of the extract was analyzed by HPLC-DAD-MS (Fig 1). Fifteen compounds (peaks 1–15) were identified in green propolis extract (Table 3). The main compounds were flavonoids and phenolic acids.

**Table 3:** Chemical compounds, mass, and retention time of compounds identified in green propolis by HPLC-DAD-MS.

Peak	*m/z*	Retention time (min)	Chemical compound	Chemical class	Structure
1	515.12	15.1	3,4-Dicafeoilquinic acid	Phenolic acid	C_25_H_24_O_12_
2	515.08	20.5	4,5-Dicafeoilquinic acid	Phenolic acid	C_25_H_24_O_12_
3	301.01	33.3	Quercetin	Flavonol	C_15_H_10_O_7_
4	230.99	50.4	3-(2,2-Dimethylchromen-6-yl) prop-2-enoic acid	Flavonol	C_14_H_14_O_3_
5	315.12	54.9	Homoferreirine	Flavonone	C_17_H_16_0_6_
6	599.023	57.5	2 [2-[4-(2 methylpropyl) phenyl] propanoyloxy] ethyl-4,5-diacetyloxy-9,10-dioxoanthracene-2-carboxylate	Anthraquinone	C_34_H_32_O_10_
7	315.12	63.7	4′,6-Dihydroxy-5,7-dimethoxy flavone	Flavonone	C_17_H_16_O_6_
8	329.17	66.3	5,7-Di-O-methylquercetin	Flavone	C_16_H_14_O7
9	487.37	68.5	Apigenin-C-hexosyl-C-deoxyexoside	Flavonoids	NI
10	299.06	70.1	3-Hydroxybiochanine A	Isoflavonones	C_16_H_12_O_5_
11	537.09	75.0	Amentoflavone	Flavonoids	C_30_H_18_O_10_
12	727.34	82.3	Trimer gallate [epi] catechin	Proanthocyanidin	NI
13	613.32	84.3	Acremoxanthone C	Xanthone	C_33_H_26_O_12_
14	491.21	96.4	Carminic acid	Anthraquinone	C_22_H_20_O_13_
15	505.25	98.6	Peonidin-3-O (6-O-acetyl)–glycoside	Glycoside	C_24_H_25_0_12_

NI: Not identified

The isolated chemical compounds, along with the retention time and observed mass, are presented in Table 3. The spectra of each peak identified by HPLC-DAD-MS are described in S1 Data. The chemical structures and masses are listed in Table 3.

### Evaluation of the antioxidant activity of green propolis extract by DPPH assay

Antioxidant activity (%) increased proportionally with extract concentration, reaching 97.99% of the maximum antioxidant activity at a concentration of 275 μg/mL. The EC_50_ value (concentration required to achieve 50% antioxidant activity) was 81.19 μg/mL.

### Phenolic compound content

The total phenolic compound content was calculated by the regression equation y = 0.006x + 0.006 (R2 = 0.999), which was obtained by using the tannic acid calibration curve (where y is the absorbance at 760 nm, and x is the concentration of tannic acid in μg/mL). The results showed that propolis extract had a total phenolic content of 135.33 mg EAT/g.

### Antifungal activity of green propolis extract (EPV) against *C*. *albicans*, *C*. *parapsilosis*, and *C*. *tropicalis*

EEPV inhibited the growth of the three tested *Candida* species (Table 4). The inhibition halo values of EEPV against the three *Candida* species are shown in Table 4. *C*. *albicans* and *C*. *tropicalis* were sensitive to the extract at 2.5 to 250 μg/mL. In contrast, *C*. *parapsilosis* was resistant to the extract at 0.25 and 2.5 μg/mL, but sensitive to the extract at 25 and 250 μg/mL.

**Table 4:** Antifungal activity of green propolis extract against *Candida* species as determined by the disk diffusion assay.

*Candida s*pecies /	Zone of inhibition (mm)				Control (AFB 16 μg/mL)
Concentrations	0.25 μg/mL	2.5 μg/mL	25 μg/mL	250 μg/mL	16 μg/mL
*C*. *albicans*	5	15.2	17.3	20.1	25
*C*. *tropicalis*	9	13.1	14.7	16.6	25
*C*. *parapsilosis*	1	6.2	10	12.1	10

### Adhesion and biofilm formation capacities of *C*. *albicans*, *C*. *tropicalis*, and *C*. *parapsilosis* on orthodontic materials (acrylic resin and steel)

**Fig 2:**
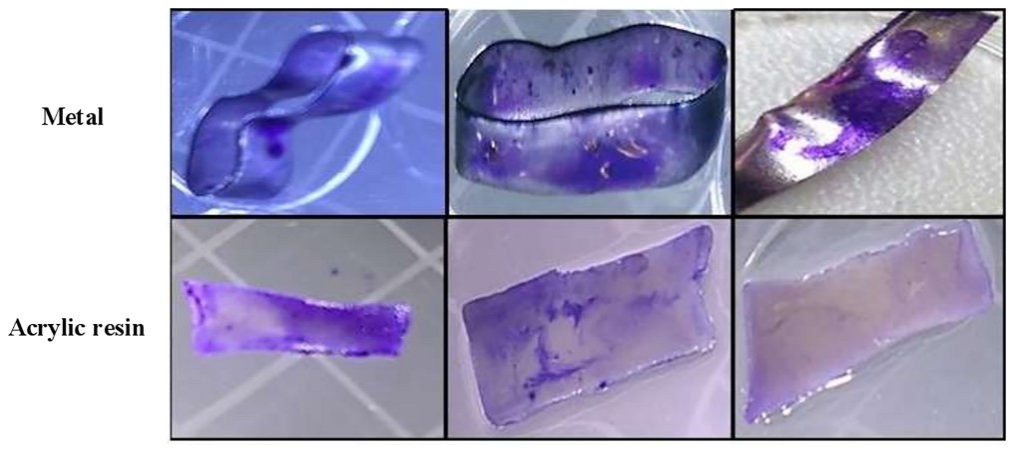
*Candida* biofilm formation on abiotic surfaces (4A: Metal and 4B: Acrylic resin) after 48 h.

All *Candida* species adhered and formed biofilms on the surfaces of the dental materials studied. On steel and resin, yeast cell adhesion affinity was weak at all incubation times, except for *C*. *albicans* at 6 and 12 h as well as C. *parapsilosis* and *C*. *tropicalis* at 12 h, which displayed moderate affinity. We observed that *C*. *albicans* showed moderate biofilm formation capacity at 24 and 48 h; *C*. *parapsilosis* showed very strong biofilm-forming propensity at 24 and 48 h; *C*. *tropicalis* displayed strong and very strong propensity at 24 and 48 h, respectively. On resin, all species displayed strong propensity at 24 and 48 h, except for *C*. *tropicalis*, which displayed very strong propensity at 48 h (Table 5 and Fig 2).

**Table 5:** Adhesion capacity and biofilm formation propensity of *C*. *albicans*, *C*. *parapsilosis*, and *C*. *tropicalis* on the surfaces of steel and acrylic resin orthodontic materials.

Time (h)	*Candida* species	Materials			
		Steel		Resin	
		Number of adherent cells	Affinity	Number of adherent cells	Affinity
3	*C*. *albicans*	351	Weak	161	Weak
	*C*. *parapsilosis*	175	Weak	178	Weak
	*C*. *tropicalis*	236	Weak	236	Weak
6	*C*. *albicans*	693	Moderate	580	Moderate
	*C*. *parapsilosis*	208	Weak	209	Weak
	*C*. *tropicalis*	262	Weak	331	Weak
12	*C*. *albicans*	1566	Strong	765	Moderate
	*C*. *parapsilosis*	459	Weak	530	Moderate
	*C*. *tropicalis*	610	Weak	520	Moderate
		Number of colonies	Propensity	Number of colonies	Propensity
24	*C*. *albicans*	331	Moderate	523	Strong
	*C*. *parapsilosis*	2435	Very strong	554.3	Strong
	*C*. *tropicalis*	913.6	Strong	945.6	Strong
48	*C*. *albicans*	349.3	Moderate	578	Strong
	*C*. *parapsilosis*	1012.3	Very strong	920	Strong
	*C*. *tropicalis*	1012.6	Very strong	2042.3	Very strong

**Fig 3:**
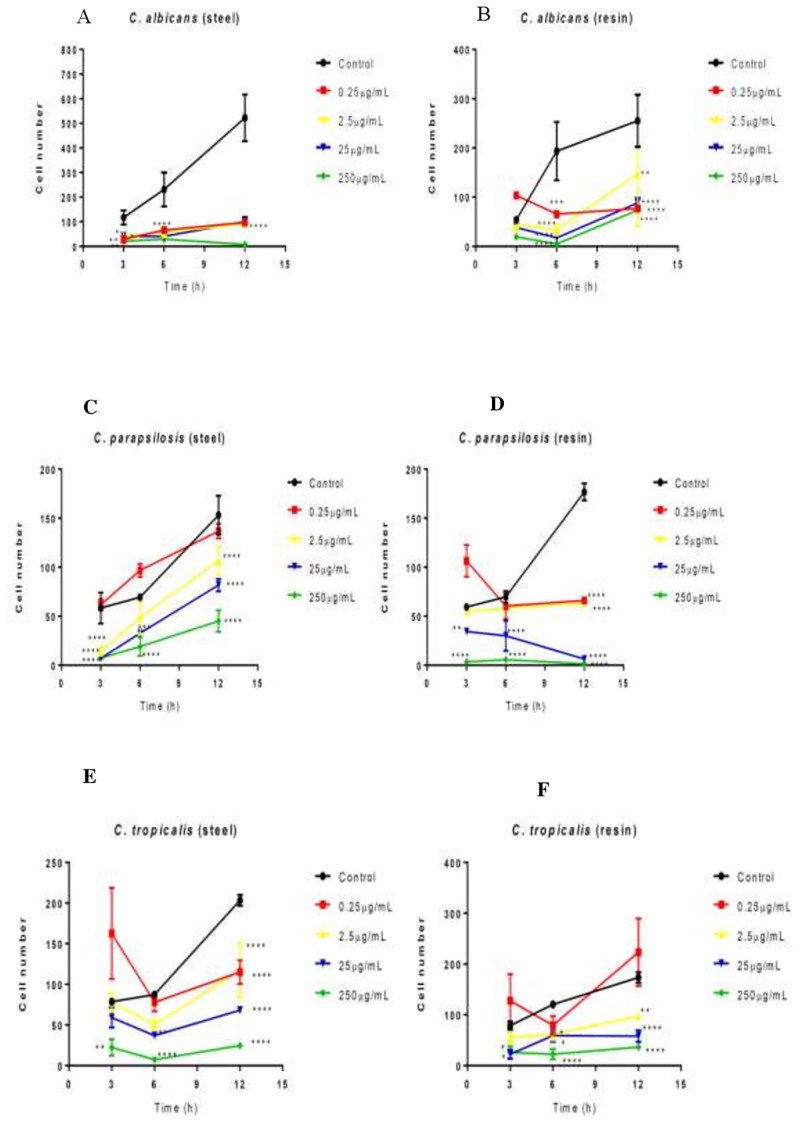
Effect of green propolis extract on the adhesion of *C*. *albicans*, *C*. *parapsilosis*, and *C*. *tropicalis* on the surfaces of dental materials (acrylic resin and steel). Effect of extract against *Candida* spp. according to time and material. *p<0.05; **p<0.01; ***p<0.001; *p<0.0001.

Fig 3 shows the antiadherence activity of hydroalcoholic green propolis extract against all *Candida* species relative to a saline control, indicating the efficient inhibition of *Candida* virulence factors by green propolis extract. All *Candida* species adhered on resin and steel, with stronger adhesion on resin. *C*. *albicans* was more sensitive to green propolis extract than the other *Candida* species.

On steel, propolis at all tested concentrations showed antiadherence effects against *C*. *albicans* at 3, 6, and 12 h (Fig 3A). On resin, green propolis extract showed antiadherence effect against *C*. *albicans* at all concentrations at 6 h (Fig 3B).

After 12 h, the extract inhibited the adhesion of *C*. *tropicalis* on steel at all concentrations tested (Fig 3E). After 3 h, the extract at 25 and 250 μg/mL was effective against adherence on resin. After 6 h, the extract at all concentrations showed antiadherence activity (Fig 3F).

**Fig 4:**
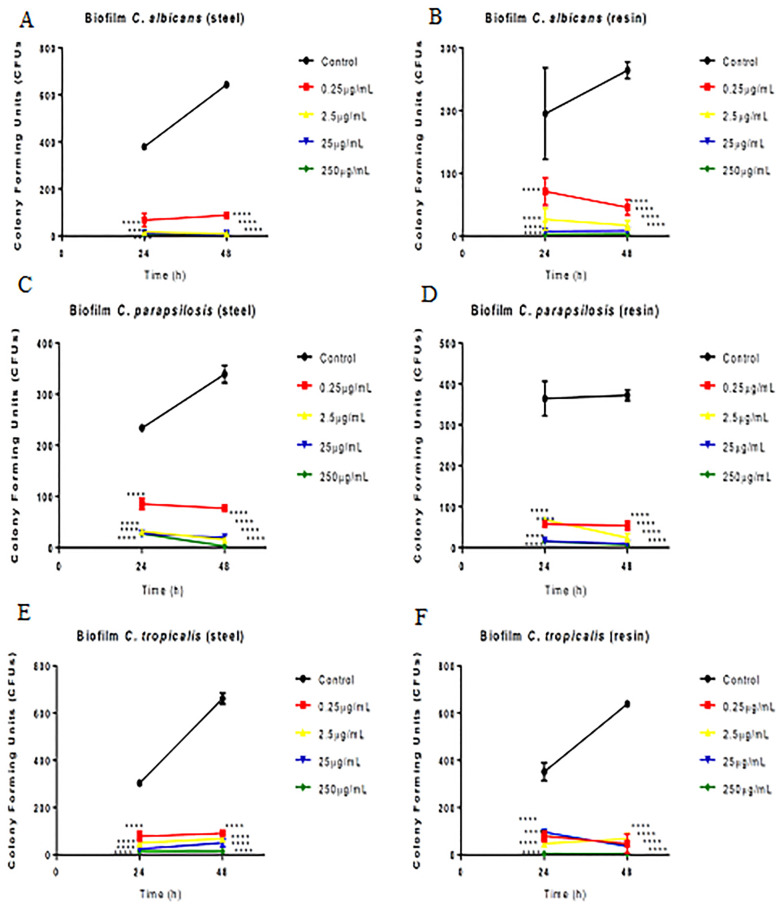
Effect of green propolis extract on biofilm formation by *C*. *albicans*, *C*. *parapsilosis*, and *C*. *tropicalis* on the surface of dental materials (acrylic resin and steel). Effect of extract against *Candida* spp. according to time and material. *p<0.05; **p<0.01; ***p<0.001; *p<0.0001.

Fig 4 shows the antibiofilm capacity of green propolis. Propolis showed significant antibiofilm activity against all *Candida* species at 24 and 48 h. All *Candida* species were able to form biofilms on steel and resin.

All the concentrations tested showed antibiofilm efficacy on both materials (steel and resin) for *C*. *albicans* (Fig 4A and 4B), *C*. *parapsilosis* (Fig 4C and 4D), and *C*. tropicalis (Fig 4E and 4F).

## Discussion

The results revealed the effective concentrations of green propolis extract against three *Candida* species. Propolis showed antiadherence and antibiofilm activities against *C*. *albicans*, *C*. *parapsilosis*, and *C*. *tropicalis* at different concentrations. The MIC of green propolis extract used in the present study against *C*. *albicans*, *C*. *parapsilosis*, and *C*. *tropicalis* was 2.5 μg/mL.

Propolis is a natural product whose composition varies with the geographic localization, climate, and utilized plant species. Antifungal activity has been previously reported for propolis. The antifungal potential of propolis is attributed to its flavonoids, especially its polyphenol and cinnamic acid content [38].

Various pharmacological properties of propolis have aroused interest in the field of dentistry owing to its potential as an antimicrobial and its efficacy in treating dental caries [39]. Propolis has been recommended by dentists as a natural therapy for maintaining oral hygiene and as an antiseptic for intracanal disinfection and treatment of oral mucositis [40].

Siqueira et al. [41] reported an antifungal MIC of 32–64 μg/mL for red propolis extract against *Candida* species isolated from chronic periodontitis patients, suggesting similar antifungal potential of green propolis extract against these yeasts, as the *Candida* species tested in this study showed sensitivity to green propolis at concentrations much lower than those reported by Siqueira et al [41].

In this study, green propolis extract showed the greatest antioxidant activity when assessed by the DPPH method. The antioxidant activity of propolis has been attributed to its high content of phenolic compounds and flavonoids [42–44].

Propolis extract has displayed excellent fungicidal and fungistatic performance in *in vitro* tests against yeasts [45]. Ota et al. showed the antifungal activity of propolis against different *Candida* species. Among these species, *C*. *albicans* was the most susceptible [46]. Siqueira et al. compared the effects of propolis and fluconazole against *Candida* species, and noted that propolis has better fungistatic and fungicidal properties than fluconazole [41].

Sforcin et al. [47] reported that *C*. *albicans* is more sensitive than *C*. *tropicalis* to propolis from São Paulo, southeastern Brazil. Similar results were obtained in this study, in which *C*. *albicans* was more sensitive to propolis than *C*. *tropicalis* (the inhibition zones formed by *C*. *albicans* were larger than those formed by *C*. *tropicalis*).

The antifungal activity of propolis against *C*. *albicans* was studied by Parcker and Luz [48] and D’Auria et al. [49], who suggested that propolis extract inhibits extracellular phospholipase activity, thus impairing fungal cell adhesion to epithelial cells. This suggestion is corroborated by the findings of the present study [50].

In this study, at all concentrations tested, propolis more strongly impaired the biofilm formation of *C*. *albicans* than that of *C*. *parapsilosis* and *C*. *tropicalis*; moreover, its effect was significantly more potent against *C*. *parapsilosis* than against *C*. *parapsilosis* (efficacy at 25 μg/mL vs 250 μg/mL for *C*. *tropicalis*), corroborating the result of Tobaldini-Valerio et al. [51], who also observed greater biofilm reduction (~ 3.5 log) in *C*. *albicans*, followed by *C*. *parapsilosis* and *C*. *tropicalis*, with log reductions of approximately 2.8 and 2, respectively, at all concentrations tested.

Similar to the results found in this study, propolis extract also showed antibiofilm activity against clinical isolates and ATCC strains of *Fusarium* species found in patients with onychomycosis, where the biomass and number of viable cells decreased significantly in the treatment group compared with those in control group [52].

Capoci et al. [35] observed a >50% reduction in CFUs for all *C*. *albicans* isolates after exposure to propolis extract compared with that in the controls. These results corroborate the findings of this study, in which reductions in CFU were observed for *C*. *albicans*, *C*. *tropicalis*, and *C*. *parapsilosis* at 25 and 250 μg/mL for all abiotic materials tested.

## Conclusions

The EEPV used in this study showed fungicidal, antiadherence, and antibiofilm activities against *C*. *albicans*, *C*. *parapsilosis*, and *C*. *tropicalis* on dental materials (steel and acrylic resin) at a concentration of 2.5 μg/mL, supporting the therapeutic use of this natural product in the treatment of oral infections by *Candida* species.

## Supporting information

S1 Data (DOCX)
