# Supplementary material for: Highly efficient antibiofilm and antifungal activity of green propolis against Candida species in dentistry materials
Source: PLoS One. 2020 Dec 23;15(12):e0228828. doi: 10.1371/journal.pone.0228828 (PMC7757894; doi:10.1371/journal.pone.0228828)
Supplement: S1 Data — (DOCX) [file pone.0228828.s001.docx]

**Supplementary data**

**Chemical compounds identified using HPLC-DAD-MS**

**
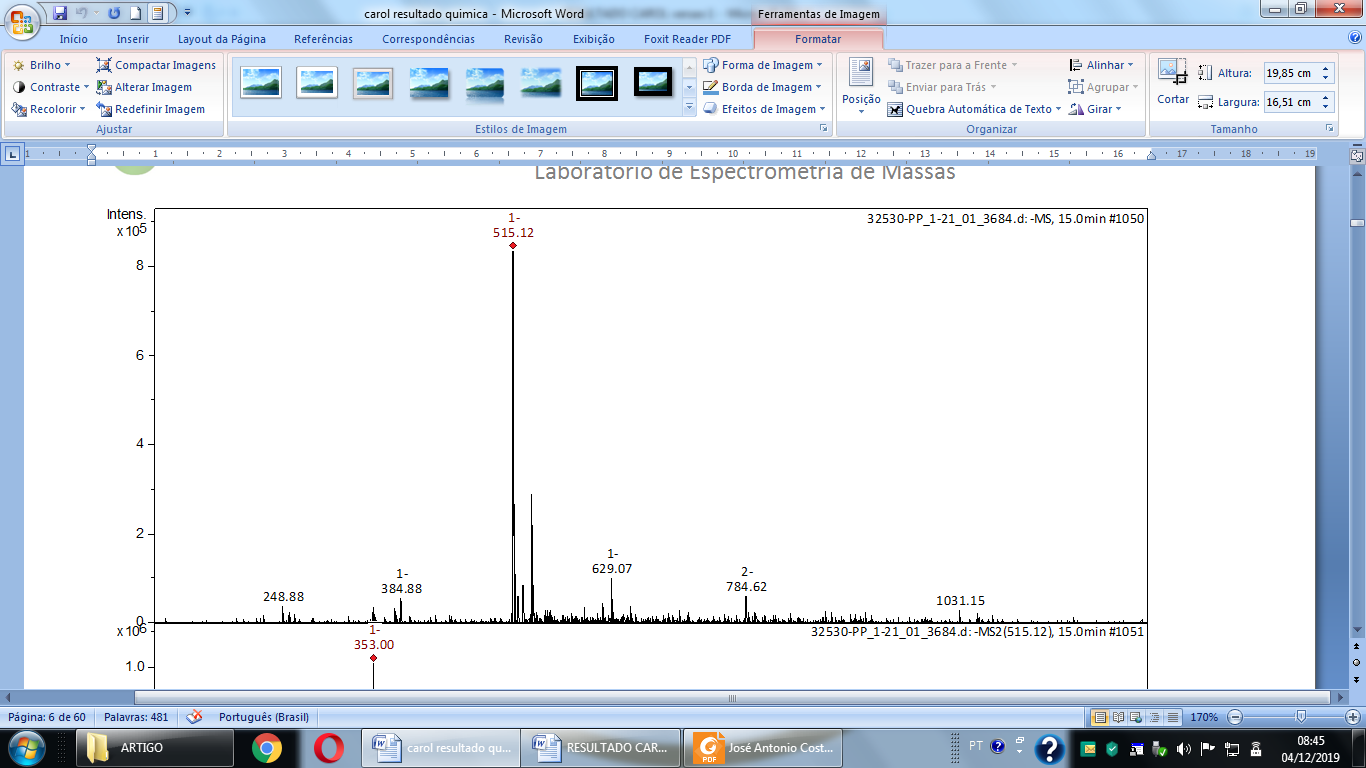
**

**Fig S1.** Mass spectrum of [M−H]^−^ for peak 1

**
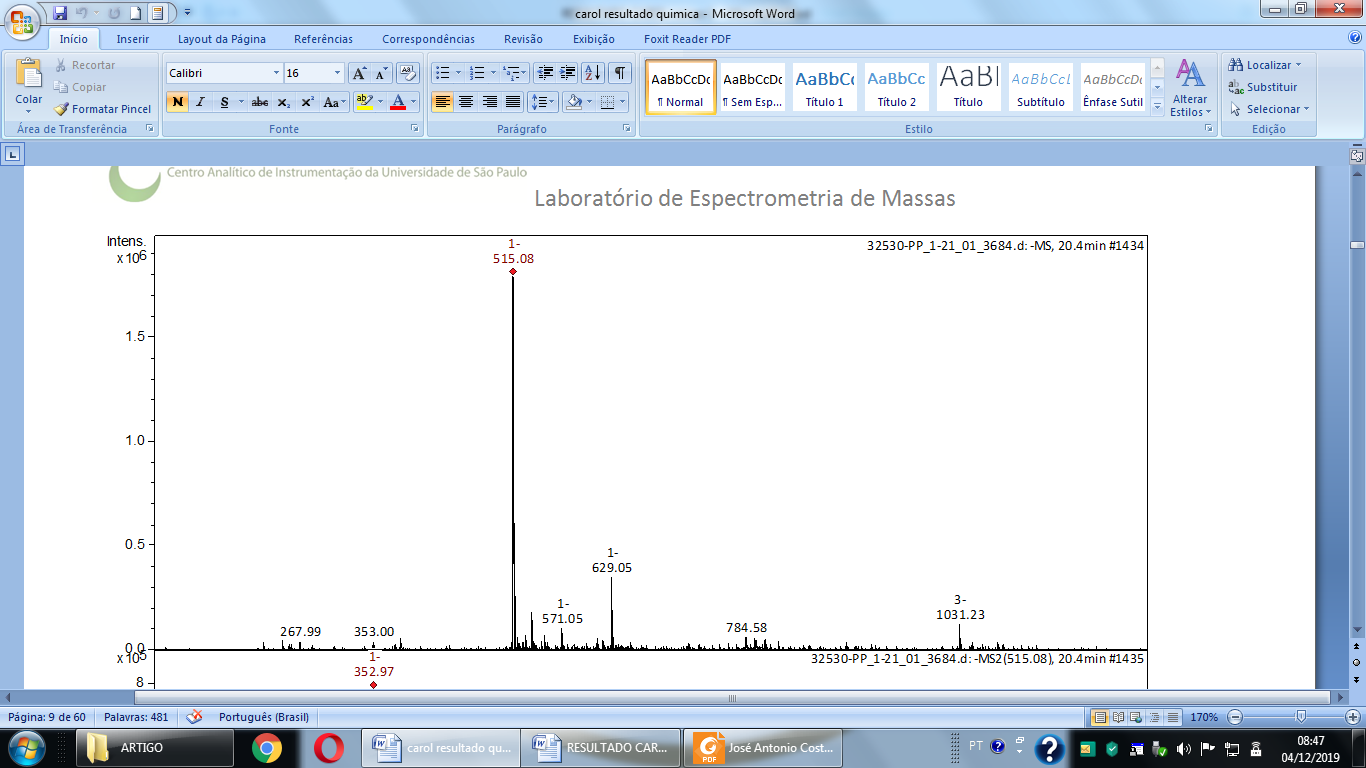
**

**Fig S2.** Mass spectrum of [M−H]^−^ for peak 2

**
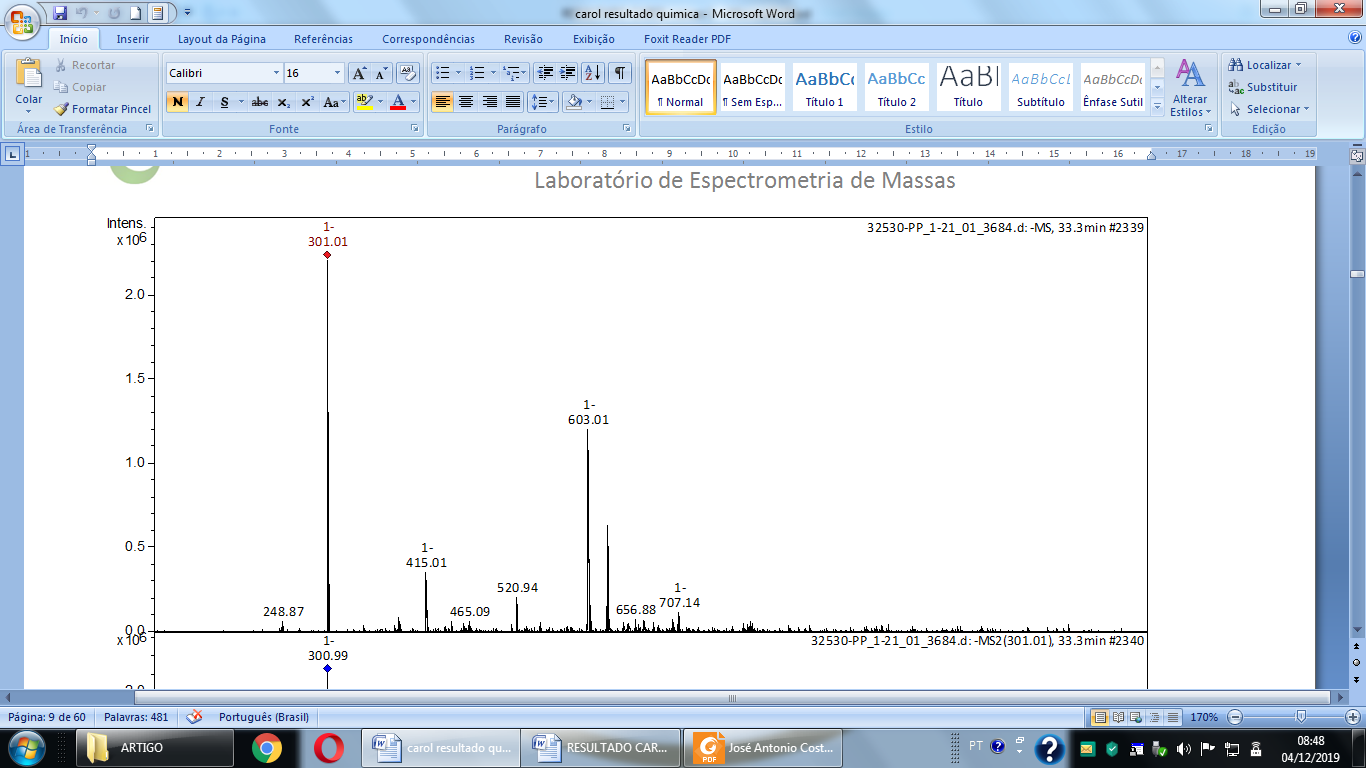
**

**Fig S3.** Mass spectrum of [M−H]^−^ for peak 3

**
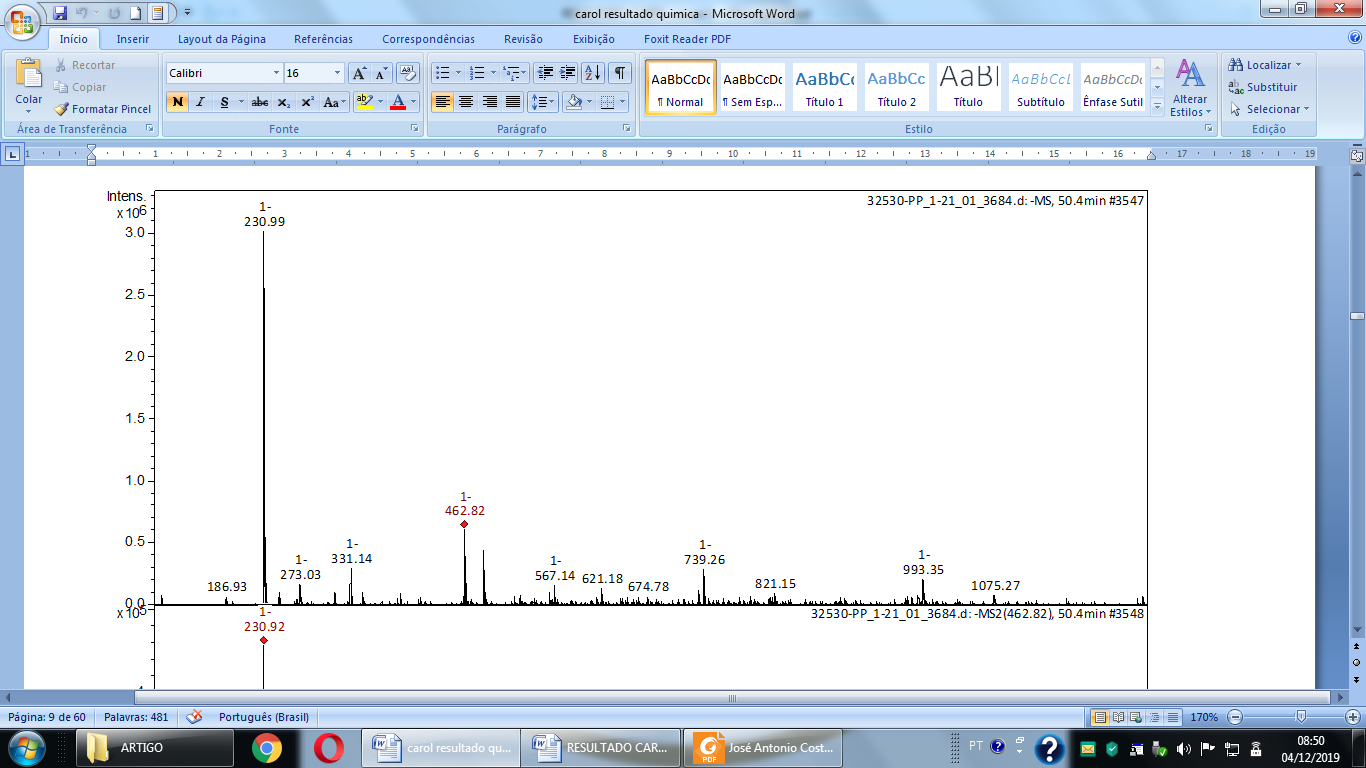
**

**Fig S4.** Mass spectrum of [M−H]^−^ for peak 4

**
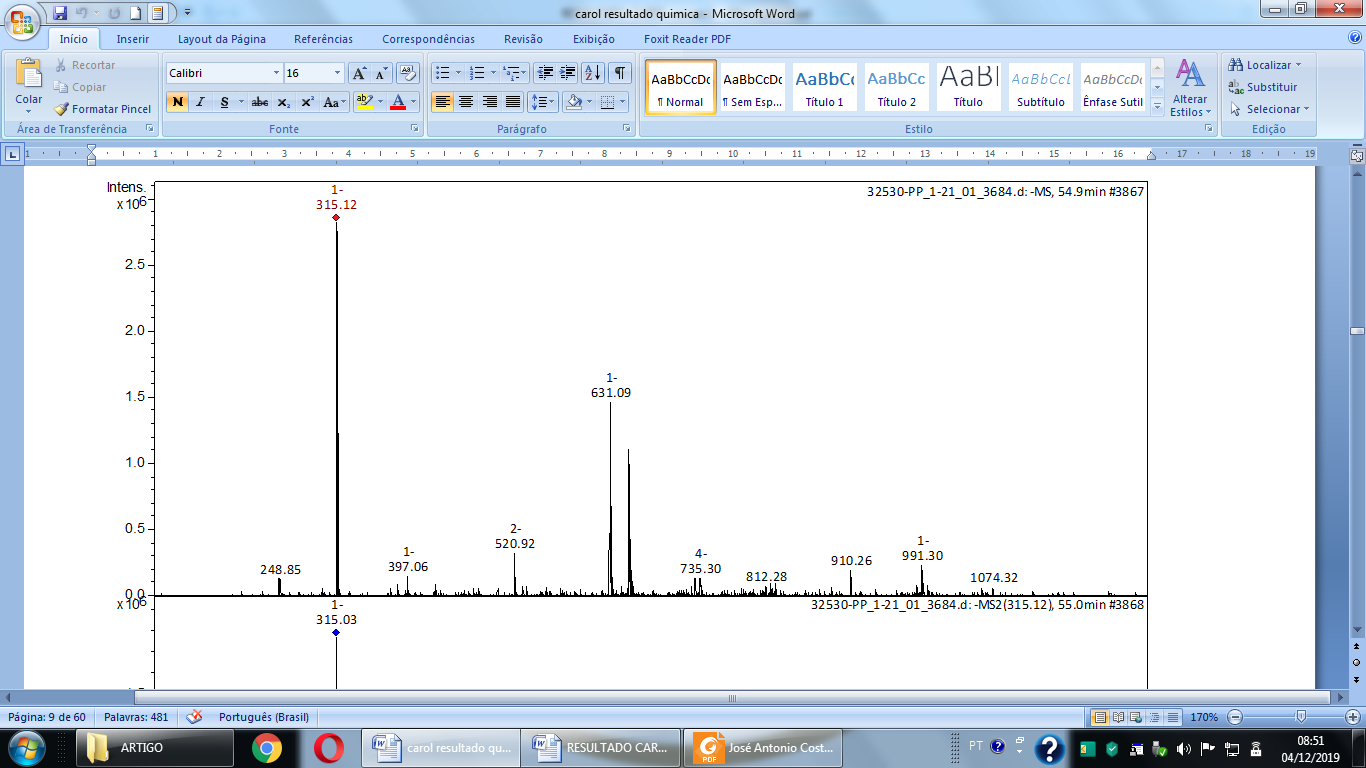
**

**Fig S5.** Mass spectrum of [M−H]^−^ for peak 5

**
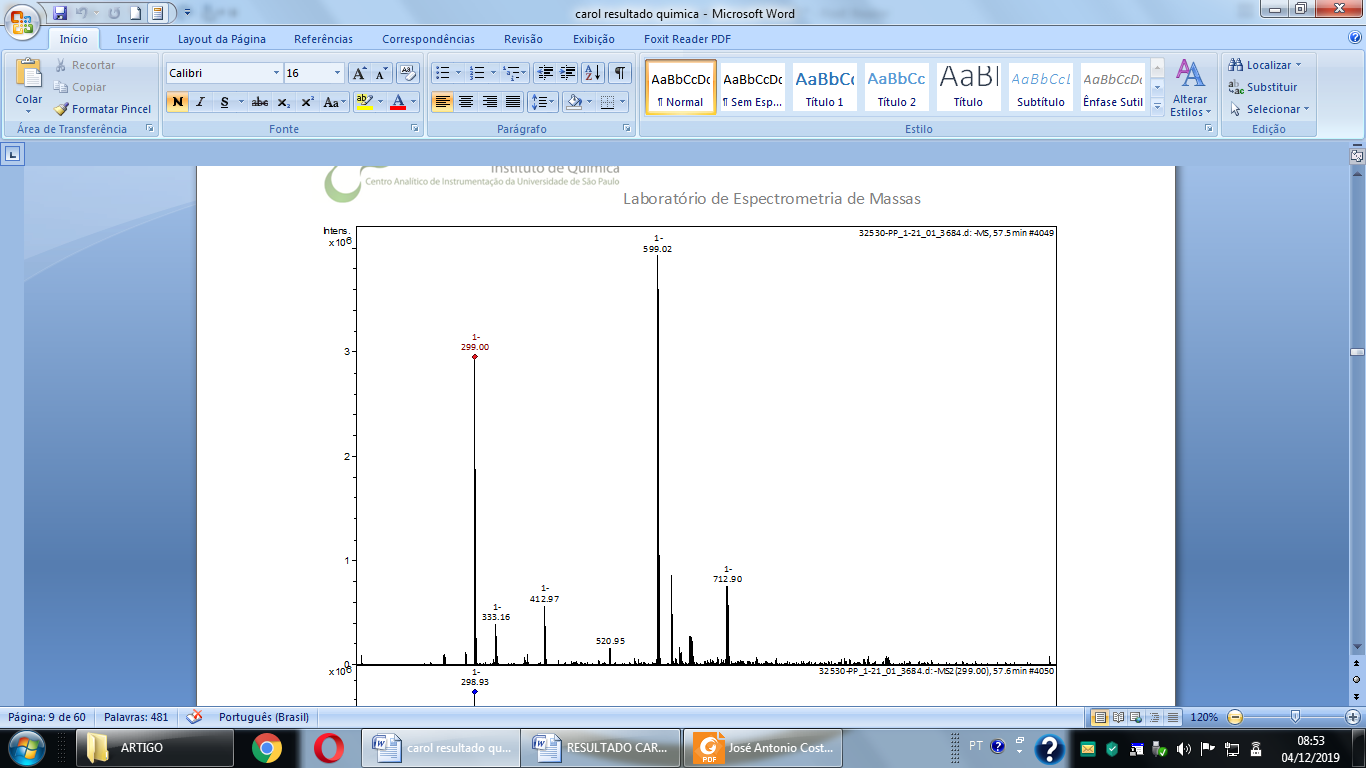
**

**Fig S6.** Mass spectrum of [M−H]^−^ for peak 6

**
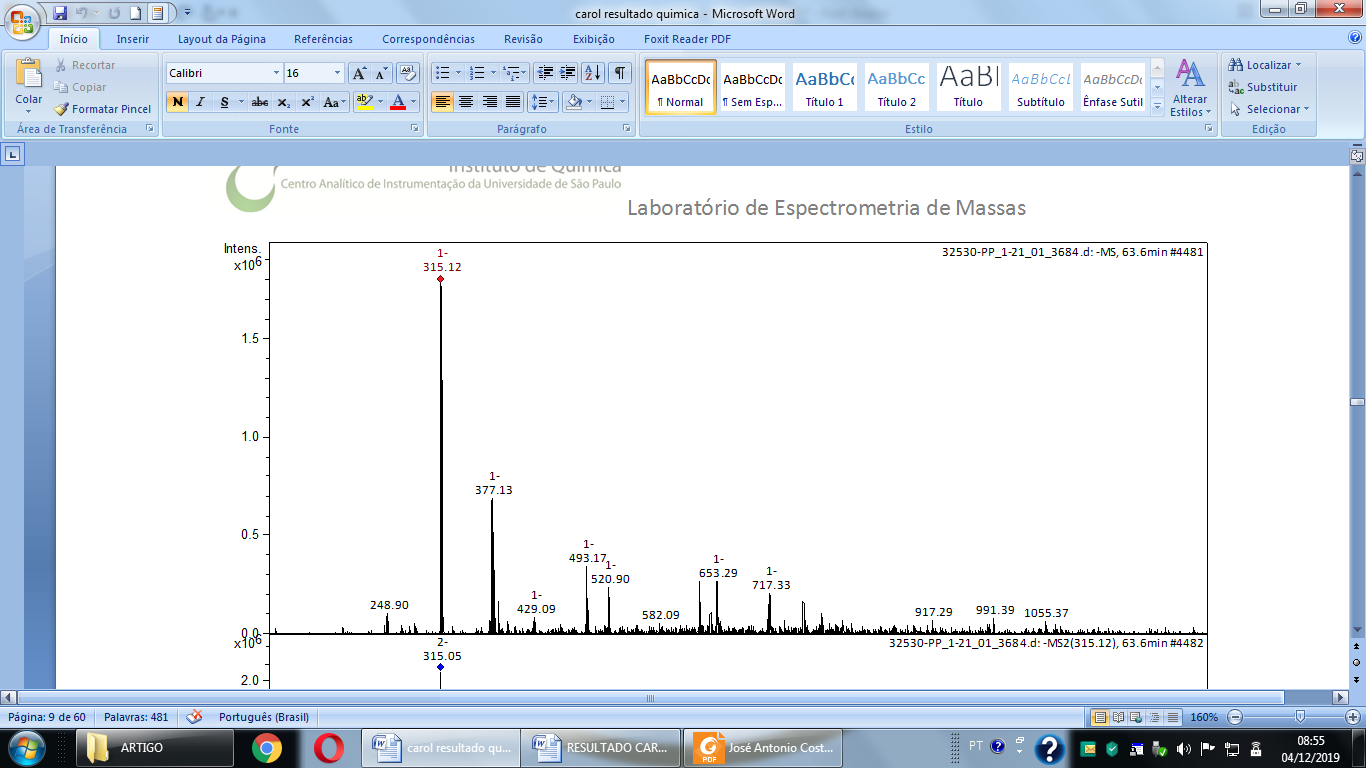
**

**Fig S7.** Mass spectrum of [M−H]^−^ for peak 7

**
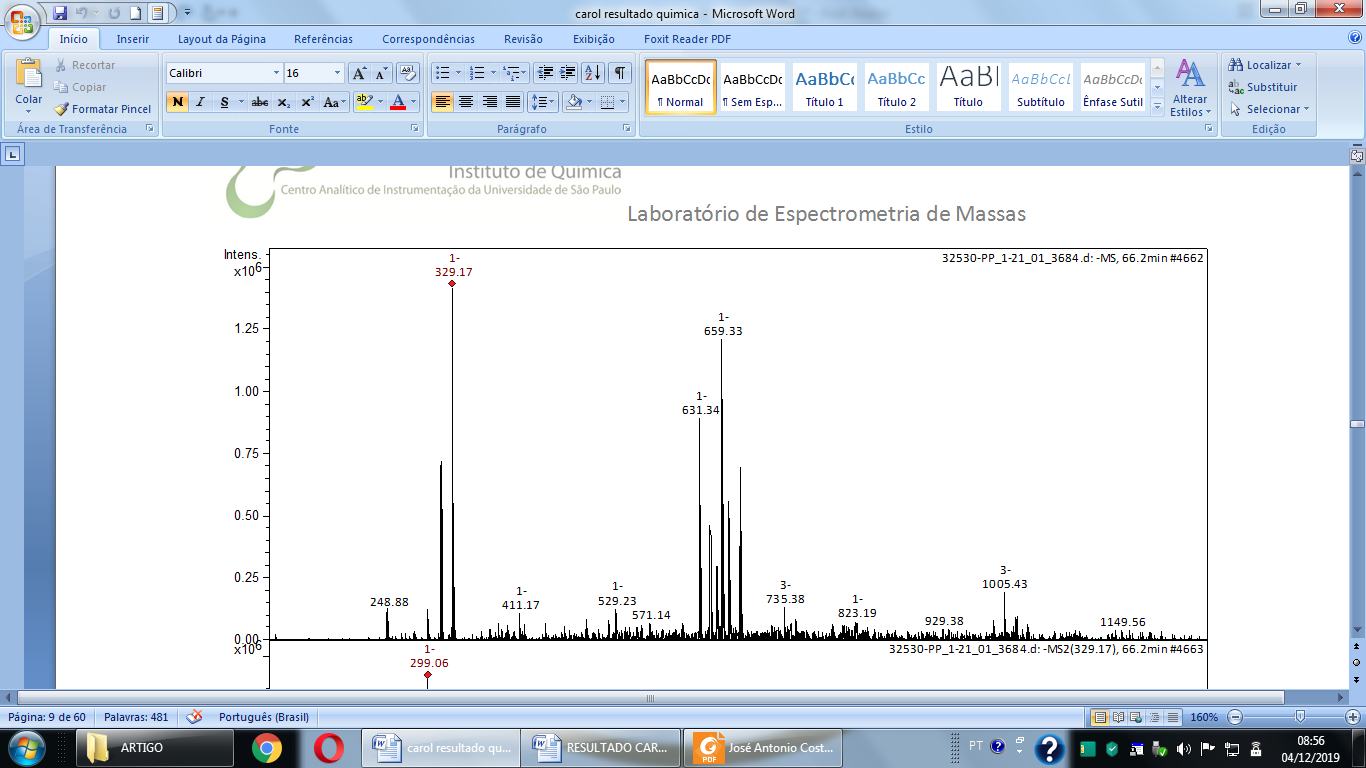
**

**Fig S8.** Mass spectrum of [M−H]^−^ for peak 8

**
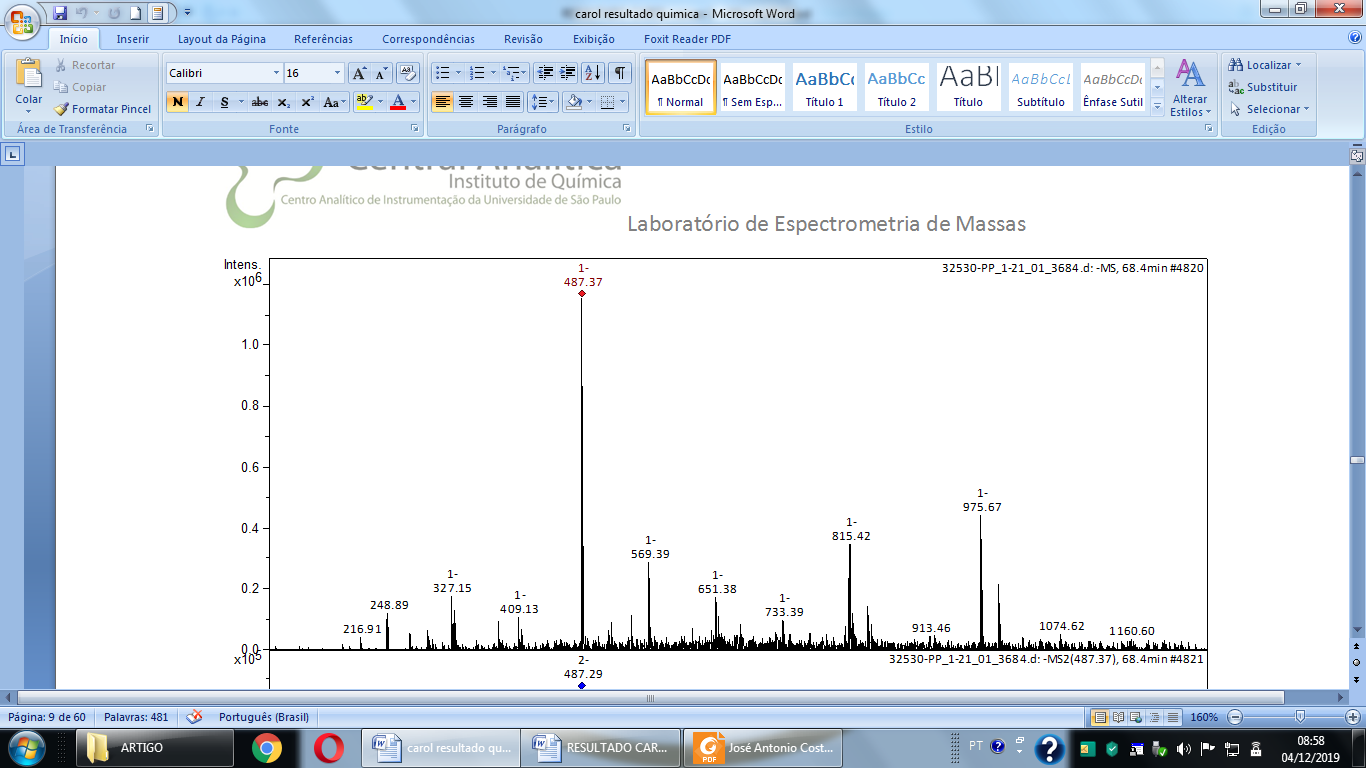
**

**Fig S9.** Mass spectrum of [M−H]^−^ for peak 9

**
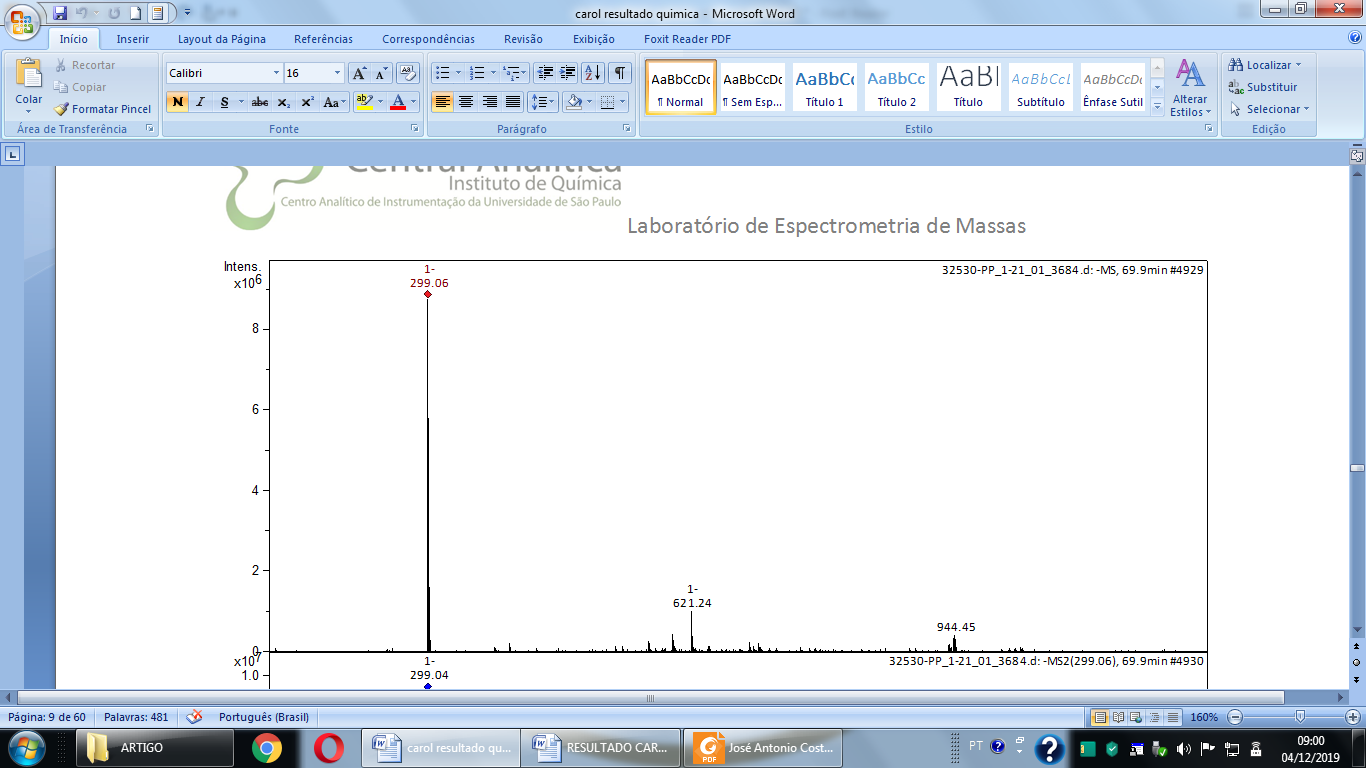
**

**Fig S10.** Mass spectrum of [M−H]^−^ for peak 10

**
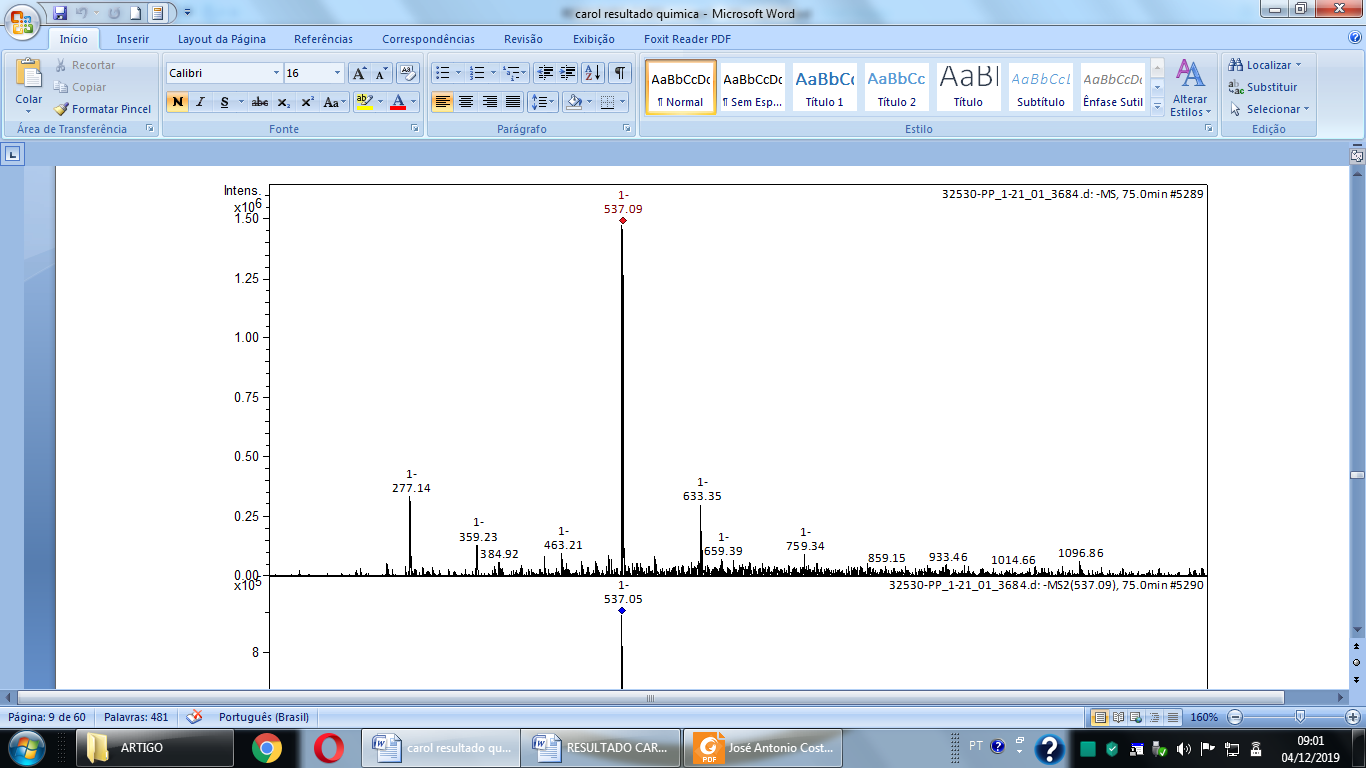
**

**Fig S11.** Mass spectrum of [M−H]^−^ for peak 11

**
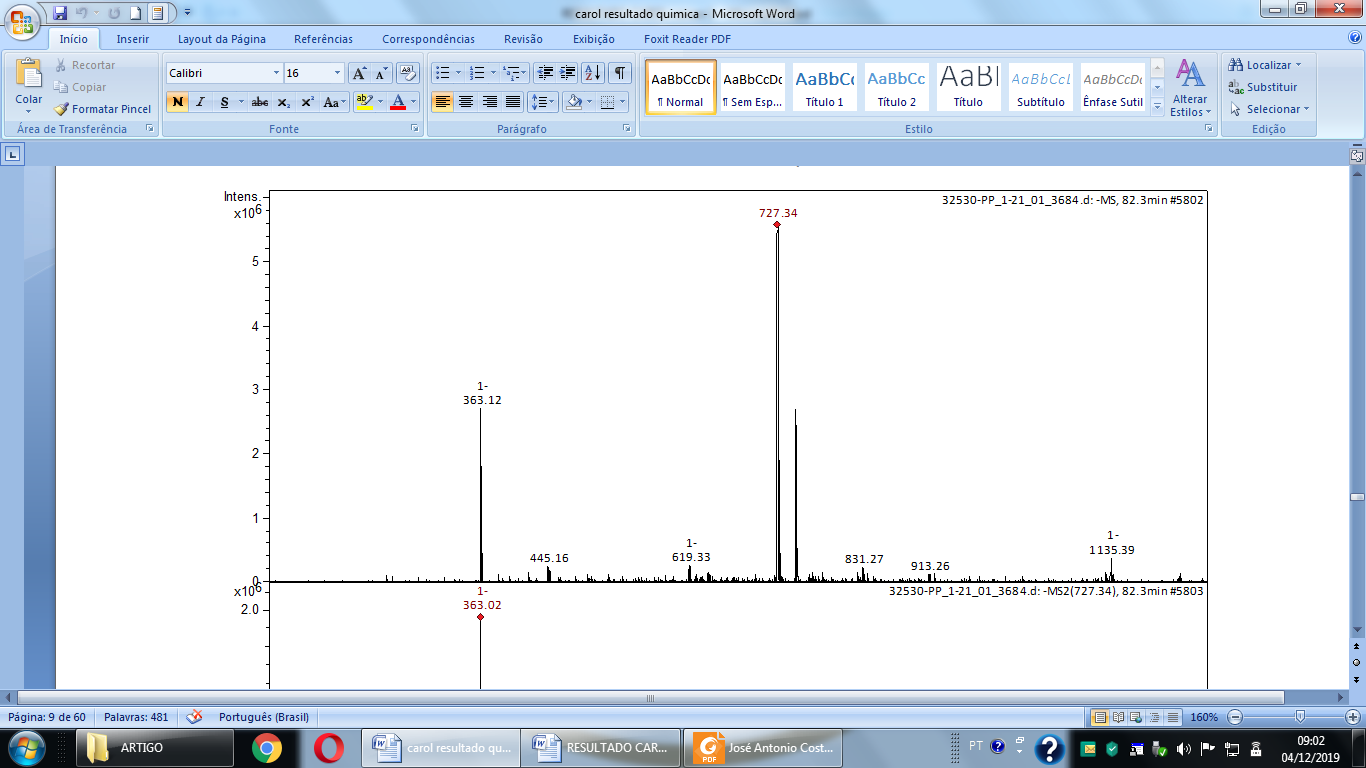
**

**Fig S12.** Mass spectrum of [M−H]^−^ for peak 12

**
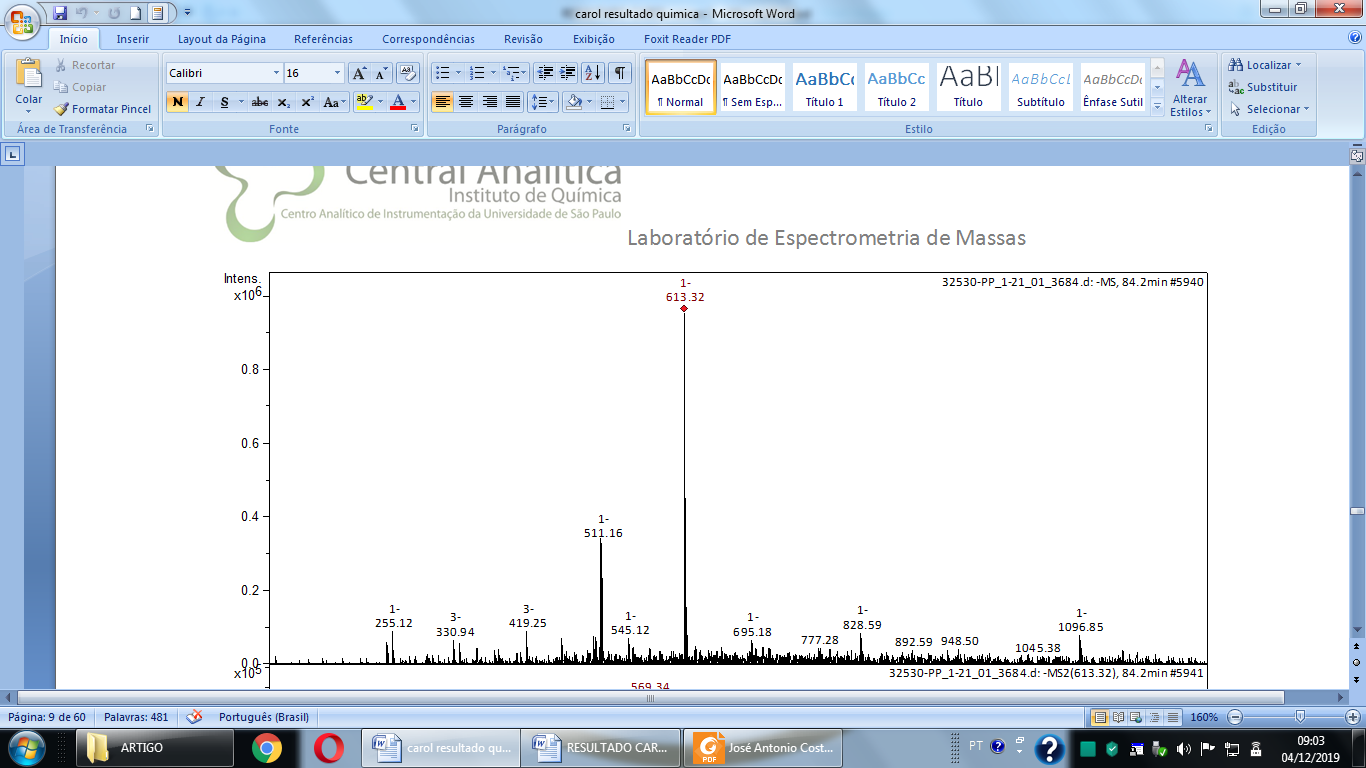
**

**Fig S13.** Mass spectrum of [M−H]^−^ for peak 13

**
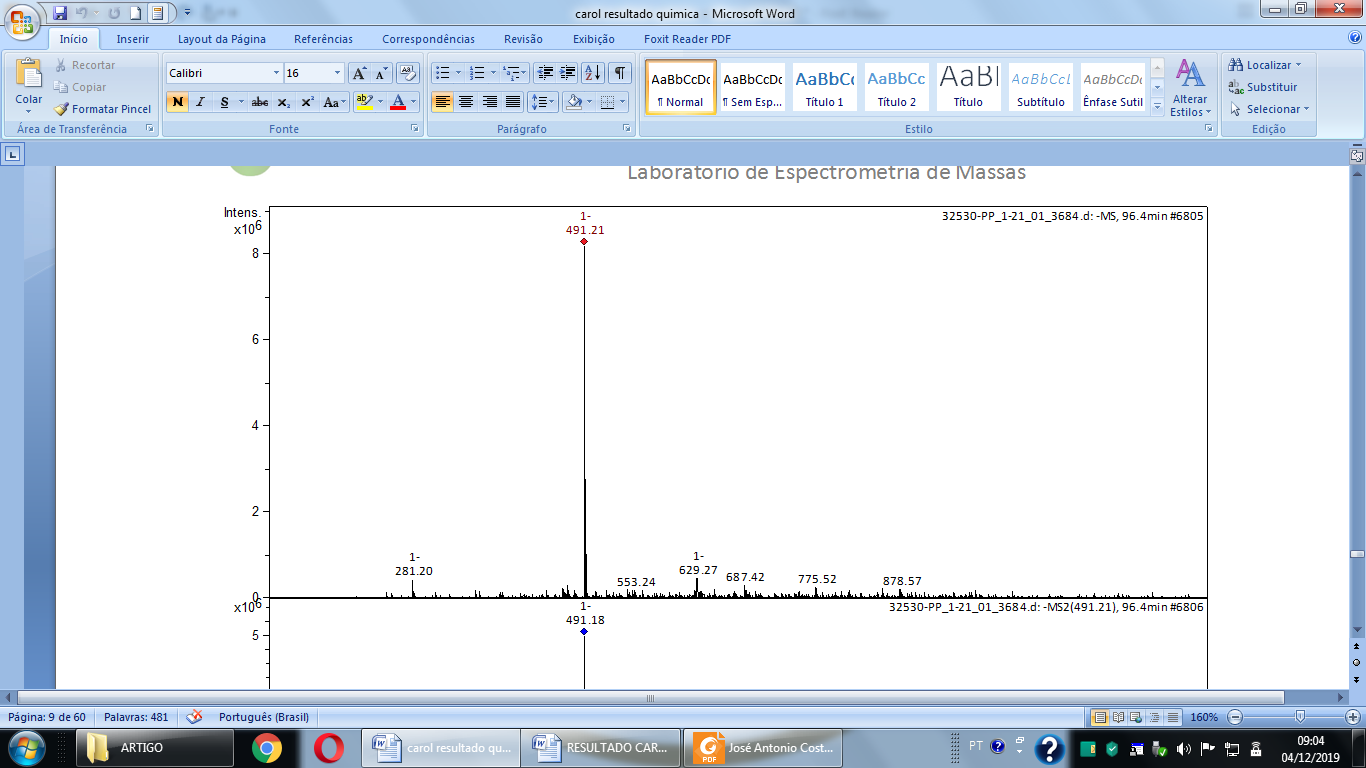
**

**Fig S14.** Mass spectrum of [M−H]^−^ for peak 14

**
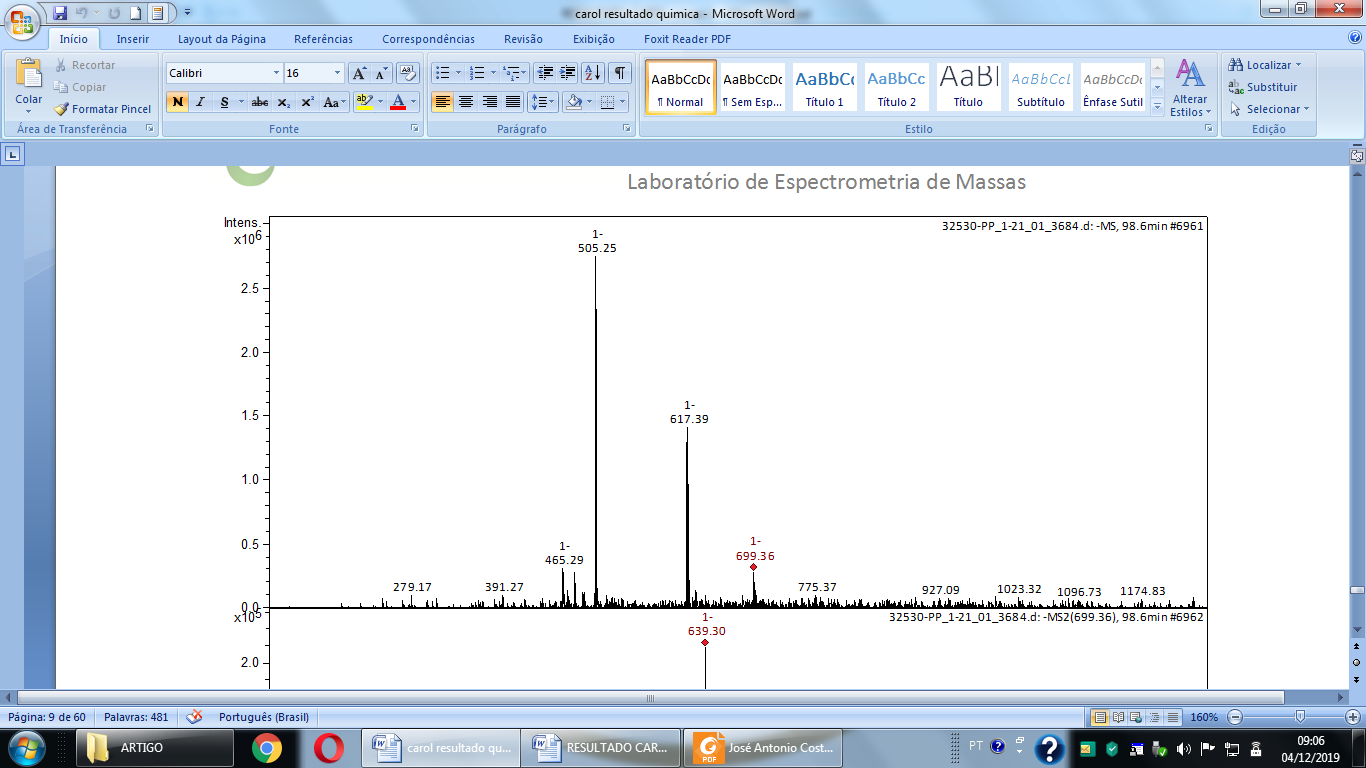
**

**Fig S15.** Mass spectrum of [M−H]^−^ for peak 15

**Antioxidant activity**


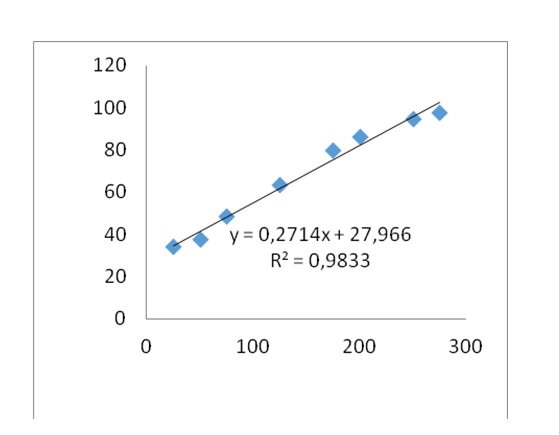


Antioxidant activity (%)

**Figure S16.** Percent antioxidant activity of hydroalcoholic green propolis extract as determined by the DPPH method.

**Phenolic content**

**
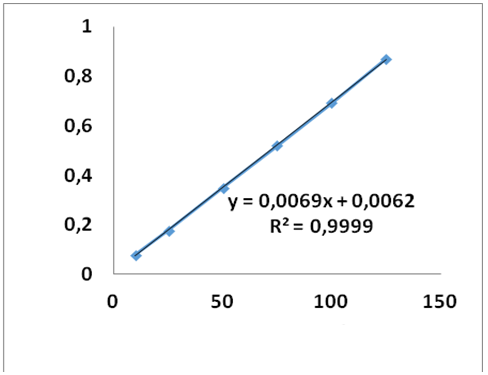
**

Concentration (µg/mL)

Absorbance

**Figure S17.** Standard curve of tannic acid for the quantification of phenolic compound content in green propolis extract.
